# Association Between Treatment of Childhood Cancer and Its Late Effects on Gonadal or Growth Function in Childhood Cancer Survivors: A Retrospective Observational Study

**DOI:** 10.1002/cam4.70805

**Published:** 2025-03-24

**Authors:** Ryuta Urakawa, Amane Noi, Hiroto Kageyama, Mikiko Ueda, Yoshiko Hashii, Kenji Ikeda

**Affiliations:** ^1^ Department of Clinical Pharmacy Research and Education, Graduate School of Pharmaceutical Sciences Osaka University Suita Osaka Japan; ^2^ Department of Pharmacy Osaka University Dental Hospital Suita Osaka Japan; ^3^ Department of Clinical Pharmacy Research and Education, School of Pharmaceutical Sciences Osaka University Suita Osaka Japan; ^4^ Graduate School of Medicine Osaka University Suita Osaka Japan; ^5^ Department of Pediatrics Osaka International Cancer Institute, Osaka Prefectural Hospital Organization Osaka Osaka Japan

**Keywords:** adverse event, chemotherapy, gonadal dysfunction, growth dysfunction, pediatric cancer, risk factor

## Abstract

**Background:**

The late effects on childhood cancer survivors have long been recognized, but detailed studies remain limited. The current study aimed to clarify the association between childhood cancer treatment and the incidence of late effects on gonadal or growth function in Japan.

**Methods:**

The retrospective study included medical records of patients with childhood cancer aged up to 15 years at diagnosis, who were treated with anticancer drugs at Osaka University Hospital from January 1, 2001, to December 31, 2019, and who survived at least 5 years after diagnosis. The patients' clinical background, treatment details, and anticancer drugs used were investigated. Multivariate logistic regression analysis was performed to assess the association between childhood cancer treatment and late effects on gonadal or growth function.

**Results:**

Of the 191 eligible patients, 83 (43.5%) developed gonadal dysfunction and 38 (19.9%) developed growth dysfunction. Multivariate logistic regression analysis showed statistically significant associations of gonadal dysfunction with female sex (odds ratio [OR], 4.79; *p* < 0.01), autologous hematopoietic stem cell transplantation (auto‐HSCT) (OR, 9.97; *p* < 0.01), allogeneic hematopoietic stem cell transplantation (allo‐HSCT) (OR, 9.48; *p* < 0.01), and platinum use (OR, 4.49; *p* = 0.037), and of growth dysfunction with auto‐HSCT (OR, 5.16; *p* < 0.01).

**Conclusion:**

Female sex, allo‐HSCT, and use of platinum are possibly associated with an increased risk of late effects on gonadal function, while auto‐HSCT may pose a risk for late effects on both gonadal and growth functions. These findings should be interpreted with caution due to the limitations of the dataset and warrant further investigation to validate these associations.

## 1 Introduction

In recent years, progress in childhood cancer treatment has been remarkable [1], and the 5‐year survival rate for childhood cancer has reached 80% [2]. However, these advancements have also heightened the importance of addressing tumor‐ or treatment‐related late effects in childhood cancer survivors (CCS). Childhood cancer is more responsive to treatment than adult cancer; therefore, strong treatment can be used. However, due to the administration of powerful treatments during the growth period, the negative effects of the treatment are significant. CCS have a higher risk of developing chronic health conditions than healthy adults. Therefore, post‐treatment monitoring and long‐term screening of CCS are increasingly being considered important [3, 4].

There are various types of late effects, such as organ dysfunction, central nervous system disorders, and secondary cancers, of which endocrine disorders constitute one of the most frequent effects, with an incidence of approximately 50% [5]. Among the endocrine disorders, late effects on gonadal or growth function cause growth or fertility problems [6, 7] and have a major impact on life plans, such as marriage and childbirth, after treatment for childhood cancer [8, 9].

The highest risk factors for growth hormone (GH) deficiency (GHD) include tumor growth or surgery within or near the hypothalamus/pituitary (HP) region, and HP radiation doses ≥ 18 Gy [10, 11, 12]. Diseases requiring hematopoietic stem cell transplantation (HSCT) after conditioning with total body irradiation (TBI) and young age during exposure to radiotherapy are additional risk factors [11, 13, 14]. Patients with late effects on gonadal function develop primary or central hypogonadism, or central precocious puberty (CPP). Risk factors for hypogonadism include radiation to the cranium, spine, abdomen, pelvis, testes, and ovaries, surgery within or near the HP region, surgical resection of the gland, and TBI for HSCT [6, 15, 16, 17, 18, 19]. In addition, alkylating agents, such as platinum, are associated with gonadal toxicity [20]. Tumors located near the hypothalamus and optic pathways, and HP exposure to radiotherapy at doses of 18–50 Gy are risk factors for CPP [6]. Although various factors have been identified as risk factors for late effects, only a few studies have comprehensively analyzed them. Because multiple risk factors have been found to overlap frequently in clinical practice, clarification of the factors should be paid more attention to by analyzing multiple factors.

Early countermeasures are required for both GHD and hypogonadism. Patients with GHD usually present with decreased linear growth velocity and develop short stature (height < −2 SD) [6]. They should be diagnosed and treated at the youngest possible age to maximize height with GH therapy before puberty. If this can be achieved, modulation of the GH dose during puberty may not be necessary [21]. Patients with hypogonadism may experience abnormal pubertal progression, male germ cell failure, primary ovarian insufficiency, and menstrual dysfunction [6, 16]. Women at risk of premature ovarian failure should not delay their childbearing age and have their ovarian reserve assessed upon consultation with a fertility specialist, as required [16]. However, young patients can face additional barriers to fertility‐preserving treatment [22, 23] due to their sexual immaturity, lack of sexual knowledge, and reluctance to visit an adult clinic or consent to egg or sperm freezing. Owing to these problems, patients and their families are often negative about fertility preservation, resulting in delays in countermeasures.

We believe that understanding the risk factors for late effects of childhood cancer treatment would enable an early response based on the patient's treatment details. In the pre‐treatment stage, knowing the risk of late effects allows patients to make informed decisions about appropriate treatment options. In the post‐treatment stage, both healthcare providers (e.g., doctors, pharmacists, etc.) and patients may be actively involved in long‐term follow‐up by gaining deeper knowledge about the risks of late effects.

Although previous studies had shown an association between childhood cancer treatment and endocrine late effects overseas [24, 25], there have been limited investigations in Japan [26, 27]. Factors that hinder the investigation of CCS include the small number of patients per facility and the loss of follow‐up during the transition from pediatrics to adult departments. For these reasons, most previous studies in Japan had only a small number of patients and were unable to perform multivariate analyses. However, Osaka University Hospital has a “long‐term follow‐up outpatient clinic,” which enables long‐term follow‐up for many CCS, including during the transition from pediatrics to adult departments. This study performed multivariate analysis on a large number of patients to assess the association between childhood cancer treatment and late effects at Osaka University Hospital. The study hypothesized that the risk of developing late effects varies with the treatment type.

## 2 Methods

### 2.1 Patients

This study included patients who met all of the following six conditions: (1) Treated with anticancer drugs from January 1, 2001, to December 31, 2019, at Osaka University Hospital, (2) under 15 years of age at the start of anticancer treatment, (3) had childhood cancer, (4) under 15 years of age at the time of diagnosis, (5) survived for at least 5 years after diagnosis, and (6) survived to be at least 15 years of age. Patients whose follow‐up period was less than 5 years due to transfer were excluded. Our study also included patients who did not undergo hormone testing or screening for hormone deficiencies. Because of the retrospective nature of the study, informed consent was waived, and the study design was approved by the Institutional Review Board of Osaka University Hospital and Osaka University Graduate School of Pharmaceutical Sciences. A description of the study, details on the opt‐out process, and an assurance that opting out would not result in any disadvantages were publicly available on the research facility's website.

### 2.2 Method of Investigation

The retrospective study considered the medical records of patients at Osaka University Hospital. Patient characteristics, such as age, sex, cancer type, details of their childhood cancer treatment, and whether they developed late effects on gonadal or growth function, were investigated. The follow‐up period was from the date of diagnosis of childhood cancer to the date of the final review of the medical records (November 1, 2021).

Childhood cancer was classified into nine types, namely leukemia, brain tumor, lymphoma, osteosarcoma, soft tissue tumor, neuroblastoma, germ cell tumor, kidney cancer, and liver cancer. Radiation sites were classified into four types, namely the head, spinal cord, abdomen and pelvis, and TBI. HSCT was classified into two types, such as autologous hematopoietic stem cell transplantation (auto‐HSCT) and allogeneic hematopoietic stem cell transplantation (allo‐HSCT). Anticancer drugs used in chemotherapy were extracted from electronic medical records and classified into the following seven types: alkylating agents, antimetabolites, anthracyclines, microtubule inhibitors, topoisomerase inhibitors, platinum, and methotrexate. Platinum agents, anthracyclines, and methotrexate, though often categorized within alkylating agents, topoisomerase inhibitors, and antimetabolites, respectively, were considered distinct categories in our analysis. Patients who developed late effects on gonadal function were defined as those who developed primary hypogonadism, central hypogonadism, or precocious puberty. We extracted physicians' diagnoses, such as primary hypogonadism, central hypogonadism, and precocious puberty from the medical records. Patients who developed late effects on growth function were defined as those diagnosed with GHD and treated with GH therapy, or those with short stature below −2.0 SD. Physicians' diagnoses, such as GHD and short stature, were extracted from medical records. In this study, “late effects” were defined as conditions that were not present at the time of cancer diagnosis or during initial treatment but developed or were diagnosed later during the follow‐up period.

### 2.3 Statistical Analysis

A chi‐squared test was performed to examine whether the incidence of late effects on gonadal or growth function differed by sex. Gonadal dysfunction was divided into primary hypogonadism, central hypogonadism, and precocious puberty, each of which was analyzed. Growth dysfunction was divided into GHD and short stature, and each was analyzed.

Logistic regression analysis was performed for the association between the treatment of childhood cancer and late effects on gonadal or growth function. Independent variables were extracted from the patients' clinical background, such as cancer type, sex, age at diagnosis (age, 0–14 years), radiation sites, HSCT, surgical treatment, anticancer drugs used, and relapse. Dependent variables included the presence or absence of late effects on gonadal or growth function. First, we analyzed the demographic models adjusted for four factors, namely cancer type, sex, age at diagnosis, and a singular treatment factor (head, spinal cord, abdomen and pelvis, TBI, others, auto‐HSCT, allo‐HSCT, surgical treatment, alkylating agents, antimetabolites, anthracyclines, microtubule inhibitors, topoisomerase inhibitors, platinum, methotrexate, others, and relapse). Next, we performed an analysis adjusting for all confounding factors, such as cancer type, sex, age at diagnosis, and all treatment factors (head, spinal cord, abdomen and pelvis, TBI, others, auto‐HSCT, allo‐HSCT, surgical treatment, alkylating agents, antimetabolites, anthracyclines, microtubule inhibitors, topoisomerase inhibitors, platinum, methotrexate, others, and relapse). Statistical analyses were performed using IBM SPSS Statistics 26. The statistical significance level was set at 5%.

## 3 Results

### 3.1 Patient Background

Three patients were excluded and 191 were included in this study. The demographic and clinical characteristics of eligible patients are shown in Table 1. The average follow‐up period was 14.9 years (range, 5–27 years). Patients included more males (53.4%) than females (46.6%), although the difference was not large. The most common type of cancer was leukemia (29.3%).

**TABLE 1:** Patient demographic and clinical characteristics.

Feature, unit	*n* = 191	
Age		
Average age at diagnosis, years (range, median)	7.39	(0–14, 8)
Average age at follow‐up, years (range, median)	22.8	(15–35)
Average period of follow‐up, years (range, median)	14.9	(5–27)
Sex		
Male, *n* (%)	102	(53.4)
Female, *n* (%)	89	(46.6)
Cancer type		
Leukemia		
Acute lymphoblastic leukemia, *n* (%)	45	(23.6)
Acute myeloid leukemia, *n* (%)	9	(4.7)
Others, *n* (%)	2	(1.0)
Lymphoma		
Non‐Hodgkin lymphoma, *n* (%)	20	(10.5)
Hodgkin lymphoma, *n* (%)	1	(0.5)
Brain tumor, *n* (%)	41	(21.5)
Osteosarcoma, *n* (%)	17	(8.9)
Soft tissue tumor, *n* (%)	15	(7.9)
Neuroblastoma, *n* (%)	13	(6.8)
Germ cell tumor, *n* (%)	9	(4.7)
Kidney cancer, *n* (%)	6	(3.1)
Liver cancer, *n* (%)	6	(3.1)
Others, *n* (%)	7	(3.7)

Patient treatment background is shown in Table 2. All 191 eligible patients were treated with chemotherapy using anticancer drugs. Treatments combined with chemotherapy included radiation therapy, HSCT, and surgery. Chemotherapy alone (24.1%) was the most common treatment. Radiation therapy included local and total body irradiation, while HSCT included bone marrow transplantation (BMT), peripheral blood stem cell transplantation (PESCT), and cord blood stem cell transplantation (CBSCT). Twenty‐eight patients received auto‐HSCT, and 47 patients received allo‐HSCT, including those who had both. Brain tumors were the most common underlying disease for autologous HSCT (50%), followed by osteosarcoma (17.9%) and liver cancer (14.3%). For allogeneic HSCT, leukemia was the most prevalent underlying disease (55.3%), with approximately 70% of cases being ALL. Among non‐leukemic conditions, neuroblastoma (12.8%), lymphoma (10.6%), and MDS (8.5%) were the most common, in that order.

**TABLE 2:** Patient treatment background.

Treatment, unit	*n* = 191	
Type of therapy		
Chemotherapy alone, *n* (%)	46	(24.1)
Chemotherapy + radiotherapy, *n* (%)	20	(10.5)
Chemotherapy + HSCT, *n* (%)	11	(5.8)
Chemotherapy + surgery, *n* (%)	27	(14.1)
Chemotherapy + radiotherapy + HSCT, *n* (%)	27	(14.1)
Chemotherapy + radiotherapy + surgery, *n* (%)	26	(13.6)
Chemotherapy + HSCT + surgery, *n* (%)	8	(4.2)
Chemotherapy + radiotherapy + HSCT + surgery, *n* (%)	26	(13.6)
Radiation sites		
Head, *n* (%)	50	(26.2)
Spinal cord, *n* (%)	15	(7.8)
Abdomen and pelvis, *n* (%)	16	(8.4)
Total body irradiation, *n* (%)	23	(12.0)
Others, *n* (%)	27	(14.1)
Hemopoietic stem cell transplantation		
Autologous hematopoietic stem cell transplantation	28	(11.0)
Allogeneic hematopoietic stem cell transplantation	47	(24.6)
Type of anticancer drug used		
Alkylating agents, *n* (%)	163	(85.3)
Antimetabolites, *n* (%)	87	(45.5)
Anthracyclines, *n* (%)	124	(64.9)
Microtubule inhibitors, *n* (%)	141	(73.8)
Topoisomerase inhibitors, *n* (%)	117	(61.3)
Platinum, *n* (%)	88	(46.1)
Methotrexate, *n* (%)	130	(68.1)
Others, *n* (%)	87	(45.5)

Abbreviation: HSCT, Hemopoietic stem cell transplantation.

### 3.2 Late Effects on Gonadal or Growth Function

The demographic and clinical characteristics of patients with late effects on gonadal or growth function are shown in Table 3. Of the 191 eligible patients, 83 (43.5%) developed late effects on gonadal function and 38 (19.9%) developed late effects on growth function. More females (56.6%) than males (43.4%) developed gonadal dysfunction. In contrast, more males (65.8%) than females (34.2%) developed growth dysfunction. Among those who developed gonadal dysfunction and those who developed growth dysfunction, the highest proportion of patients received chemotherapy + radiotherapy + HSCT + surgery.

**TABLE 3:** Demographic and clinical characteristics of patients with gonadal or growth dysfunction.

	Total	Gonadal dysfunction		Growth dysfunction	
	*n* = 191	*n* = 83		*n* = 38	
Sex					
Male, *n* (%)	102	36	(43.4)	25	(65.8)
Female, *n* (%)	89	47	(56.6)	13	(34.2)
Treatment					
Chemotherapy alone, *n* (%)	46	5	(6.0)	2	(5.3)
Chemotherapy + radiotherapy, *n* (%)	20	10	(12.1)	5	(13.2)
Chemotherapy + HSCT, *n* (%)	11	4	(4.8)	4	(10.5)
Chemotherapy + surgery, *n* (%)	27	6	(7.2)	0	(0)
Chemotherapy + radiotherapy + HSCT, *n* (%)	27	20	(24.1)	9	(23.7)
Chemotherapy + radiotherapy + surgery, *n* (%)	26	9	(10.8)	3	(7.9)
Chemotherapy + HSCT + surgery, *n* (%)	8	6	(7.2)	4	(10.5)
Chemotherapy + radiotherapy + HSCT + surgery, *n* (%)	26	23	(27.7)	11	(28.9)

Abbreviation: HSCT, hemopoietic stem cell transplantation.

The results of the chi‐squared test for sex differences in the incidence of late effects on gonadal or growth function are shown in Table 4. The results indicated a sex difference in patients who developed late effects on gonadal function (*p* < 0.05) and in those who developed central hypogonadism (*p* < 0.01).

**TABLE 4:** Sex differences in the incidence of gonadal or growth dysfunction.

	Female	Male	Total	*p*
	*n* = 89	*n* = 102	*n* = 191	
Late effects on gonadal function	47	36	83	0.015*
Primary hypogonadism	31	28	59	0.271
Central hypogonadism	15	5	20	0.007*
Precocious puberty	1	3	4	0.382
Late effects on growth function	13	25	38	0.087
Growth hormone deficiency	5	7	12	0.724
Short stature (< −2.0SD)	8	18	26	0.082

*Note:* Chi‐squared test was performed.

* *p* < 0.05.

### 3.3 Association Between Treatment and Late Effects

The results of the logistic regression analysis for the association between childhood cancer treatment and late effects on gonadal or growth function are shown in Table 5. The results of the fully adjusted models showed statistically significant associations of late effects on gonadal function with female sex (odds ratio [OR], 4.79; 95% confidence interval [CI], 1.93–11.94, *p* < 0.01), auto‐HSCT (OR, 9.97; 95% CI, 2.46–40.42; *p* < 0.01), allo‐HSCT (OR, 9.48; 95% CI, 1.88–47.74; *p* < 0.01), and platinum use (OR, 4.49; 95% CI, 1.10–18.37; *p* = 0.037), and of late effects on growth function with auto‐HSCT (OR, 5.16; 95% CI, 1.50–17.80; *p* < 0.01).

**TABLE 5:** Association between treatment and gonadal or growth dysfunction.

	Gonadal dysfunction		Growth dysfunction	
	OR	(95% CI)	OR	(95% CI)
Demographic adjusted models				
Radiation sites				
Head	2.61	(1.27–5.40)**	3.57	(1.52–8.37)**
Spinal cord	2.57	(0.82–8.00)	2.52	(0.77–8.20)
Abdomen and pelvis	2.57	(0.82–8.05)	1.23	(0.30–5.18)
Total body irradiation	11.81	(3.56–39.16)**	3.62	(1.34–9.76)*
Others	2.24	(0.94–5.35)	1.49	(0.53–4.23)
HSCT				
Autologous HSCT	8.17	(2.85–23.37)**	4.19	(1.65–10.66)**
Allogeneic HSCT	5.32	(2.50–11.31)**	3.13	(1.44–6.80)**
Surgical treatment	1.91	(0.98–3.69)	1.55	(0.65–3.72)
Type of anticancer drug used				
Alkylating agents	3.52	(1.32–9.39)*	1.60	(0.51–5.03)
Antimetabolites	0.72	(0.38–1.38)	0.96	(0.42–2.20)
Anthracyclines	1.03	(0.56–1.91)	0.39	(0.19–0.84)*
Microtubule inhibitors	1.12	(0.57–2.21)	0.43	(0.19–0.95)*
Topoisomerase inhibitors	7.38	(3.52–15.48)**	1.68	(0.76–3.70)
Platinum	4.07	(2.08–7.97)**	2.30	(1.01–5.24)*
Methotrexate	1.55	(0.73–3.26)	2.21	(0.80–6.16)
Others	1.05	(0.58–1.90)	0.47	(0.21–1.02)
Relapse	2.49	(1.18–5.25)*	2.88	(1.29–6.41)**
Fully adjusted models				
Cancer type	0.98	(0.79–1.22)	1.01	(0.79–1.28)
Sex	4.79	(1.93–11.94)**	0.51	(0.19–1.33)
Age at diagnosis	1.01	(0.91–1.11)	0.92	(0.83–1.03)
Radiation sites				
Head	1.30	(0.40–4.20)	2.61	(0.71–9.58)
Spinal cord	0.71	(0.12–4.04)	0.60	(0.11–3.15)
Abdomen and pelvis	0.87	(0.15–5.18)	0.63	(0.09–4.66)
Total body irradiation	5.00	(0.90–27.96)	4.74	(0.89–25.16)
Others	1.25	(0.41–3.83)	1.55	(0.41–5.82)
HSCT				
Autologous HSCT	9.97	(2.46–40.42)**	5.16	(1.50–17.80)**
Allogeneic HSCT	9.48	(1.88–47.74)**	2.76	(0.53–14.30)
Surgical treatment	0.62	(0.21–1.83)	0.89	(0.29–2.76)
Type of anticancer drug used				
Alkylating agents	1.06	(0.24–4.70)	0.91	(0.18–4.61)
Antimetabolites	0.40	(0.08–2.08)	1.45	(0.30–7.03)
Anthracyclines	1.30	(0.38–4.40)	0.56	(0.14–2.27)
Microtubule inhibitors	1.68	(0.60–4.72)	0.70	(0.24–2.04)
Topoisomerase inhibitors	2.41	(0.89–6.50)	0.53	(0.14–1.97)
Platinum	4.49	(1.10–18.37)*	2.42	(0.47–12.45)
Methotrexate	1.31	(0.36–4.83)	1.54	(0.35–6.71)
Others	1.16	(0.39–3.45)	0.55	(0.13–2.31)
Relapse	1.32	(0.45–3.82)	2.10	(0.76–5.81)

*Note:* Logistic regression analysis was performed. The demographic‐adjusted models were adjusted for cancer type, sex, age at diagnosis, and respective factors. The fully adjusted models were adjusted for all confounding factors.

Abbreviations: CI, confidence interval; HSCT, hemopoietic stem cell transplantation; OR, odds ratio.

* *p* < 0.05.

** *p* < 0.01.

## 4 Discussion

Previous studies, conducted overseas, on the association between childhood cancer treatment and late effects had revealed that radiation therapy and alkylating agents are risk factors for gonadal dysfunction [15, 16]. Examples of radiation therapy include radiation to the cranium, spine, abdomen, and pelvis; examples of alkylating agents include cyclophosphamide, ifosfamide, and busulfan [17, 18, 28, 29]. Cranial irradiation is a risk factor for growth dysfunction [30]. In recent years, including in Japan, studies have been conducted to clarify the risk factors for late effects in childhood cancer treatment, and similar results have been reported in previous overseas studies as well [26]. However, most of these studies had limitations in terms of small study populations, and multivariate analysis could not be performed. Therefore, in this study, we performed a multivariate logistic regression analysis considering multiple factors in 191 CCS to clarify the association between childhood cancer treatment and late effects.

Multivariate logistic regression analysis revealed platinum, auto‐ and allo‐HSCT, and female sex to be significant factors that cause late effects on gonadal function. Platinum compounds, such as carboplatin and cisplatin, are drugs associated with intermediate risk for gonadotoxicity [31]. The drugs are also risk factors for gonadal dysfunction, according to the Long‐Term Follow‐Up Guidelines of the Children's Oncology Group 20; the results of our study supported the same. Approximately two‐thirds (females, 90%–99%; males, 60%–90%) of patients who received auto‐HSCT and allo‐HSCT developed gonadal impairment early [32]. In addition, the OR of auto‐ and allo‐HSCT for late effects on gonadal function was the highest (auto‐HSCT: OR, 9.97; allo‐HSCT: OR, 9.48), based on the multivariate logistic regression analysis. Taken together, we suggested that HSCT is a significant factor in the development of late effects on gonadal function. In female CCS, hypogonadism has long been a problem, and the risk of premature nonsurgical menopause is approximately 13.2 times that in their siblings [33]. In female CCS with childhood lymphoma, the hypogonadism rate (44%) is approximately four times higher than males (11%), but more females than males had achieved post‐treatment parenthood (*p* < 0.001) [34]. This fact suggested that gonadal dysfunction and actual infertility may not be a perfect match. In addition, spermatogonia are highly sensitive to the cytotoxic effects of radiation and anticancer drugs, and 15%–30% of male cancer survivors lose their fertility [35]. Logistic regression analysis, in our study, found that females may be more likely to develop late effects on gonadal function than males in all CCS (OR, 4.79; *p* < 0.01). The chi‐square test in our study found that central hypogonadism, in particular, may contribute to gender differences (*p* < 0.01). Further research would be required on sex differences in gonadal toxicity and the risk of developing infertility.

Our study further revealed that auto‐HSCT is the most significant factor that causes late effects on growth function. In previous studies, patients who received HSCT had a higher risk of developing problems in growth and physical function compared to those receiving chemotherapy alone [36]. In patients receiving auto‐HSCT, serum IGF‐I levels were below the age‐reference values within 3 months after transplantation, with approximately 20% returning to the normal range and approximately 38% remaining low up to 1 year after transplantation [32]. These facts suggested that auto‐HSCT could be a significant factor that causes late effects on growth function, as also seen in our study. Although our study did not find a statistically significant association with allo‐HSCT, patients who received allo‐HSCT did develop acute or chronic graft‐versus‐host disease (GVHD), and the latter could cause growth retardation [32]. Therefore, further research considering GVHD would be required in the future.

The strengths of this study included the multivariate logistic regression analysis with multiple factors, including treatments (auto‐ and allo‐HSCT and surgical treatment), radiation sites (four types), anticancer drugs used (seven types), and relapse. Previous studies have indicated that radiation therapy and alkylating agents are risk factors for late effects on gonadal function, and cranial irradiation is a risk factor for late effects on growth function [15, 16, 30]. However, no previous study had performed multivariate logistic regression analysis using as many factors as our study to investigate the association between childhood cancer treatment and late effects. In this study, multivariate analysis showed that female sex, platinum, and both auto‐ and allo‐HSCT were the most significant risk factors for developing late effects on gonadal function, with auto‐HSCT being significantly associated with late effects on growth function.

The current study had some limitations. First, selection bias possibly exists because it was a retrospective study conducted in a single hospital, which may provide more advanced medical care and may have a higher severity of the primary disease. Therefore, a multicenter prospective study is recommended to obtain universal results in Japan. Second, the follow‐up period was short, with an average of 14.9 (range, 5–27) years. Since the incidence of late effects increases over time [3, 37], additional late effects may develop in the future. Therefore, the study should be continued, and data should be analyzed over a longer follow‐up period. Third, we extracted physicians' diagnoses from the medical records and used them as definitions of late effects on gonadal and growth function. Thus, the study lacks information on the evaluation methods and quantitative criteria used in the diagnosis. Finally, because of the technical issues in data extraction of electronic health records, the study lacks information on radiation and anticancer drug doses, the combination of chemotherapeutic agents such as the conditioning regimen for HSCT, and GVHD, all of which are risk factors for gonadal or growth dysfunction [16, 32, 38, 39]. Cyclophosphamide equivalent dose (CED) can be used to quantify alkylating agent exposure [40], which was not used in this study. Therefore, further studies should be conducted with more such information to survey items.

The results of our study can provide useful information for better medical care in both pre‐treatment and post‐treatment follow‐up stages. Female sex, platinum use, and allo‐HSCT are risk factors for developing late effects on gonadal function, and auto‐HSCT is a risk factor for developing late effects on gonadal and growth function. Therefore, a more careful follow‐up would be required for CCS with these risk factors. In the pre‐treatment stage, doctors can explain the risk of late effects on gonadal function in advance so that patients can actively consult with their doctor about fertility‐preserving treatment, such as egg or sperm freezing. In the post‐treatment follow‐up stage, doctors should deepen their knowledge about late effects and understand the risk factors in advance, which can in turn enable them to formulate plans for examinations and treatments according to the risk factors of the patients. Patients' sense of urgency about the risk of late effects could become stronger after receiving explanations from doctors, and they may be expected to actively participate in long‐term follow‐ups, such as visiting the adult department even at an early age, undergoing egg or sperm freezing, and continuing to visit the hospital for a long time without interruption. Overall, the results of this study provide new knowledge for deepening the understanding of both healthcare providers and patients, thereby leading to prevention, early detection, and treatment of late effects.

## 5 Conclusions

Our study aimed to assess the risk of late effects on gonadal and growth function associated with different childhood cancer treatments. While our findings suggest that factors such as female sex, platinum‐based therapies, and HSCT are associated with increased risks, we acknowledge the limitations of our dataset and the need for cautious interpretation of our results. Future studies with larger, more diverse cohorts and detailed treatment data are necessary to validate these findings and clarify the relationship between specific treatments and late effects. In Japan, long‐term follow‐up studies on childhood cancer survivors remain limited. We hope our work encourages further research in diverse regions and supports the evolution of childhood cancer treatment strategies with greater consideration for late effects.

## Author Contributions

**Ryuta Urakawa:** conceptualization (equal), data curation (equal), formal analysis (lead), investigation (equal), methodology (lead), project administration (equal), resources (equal), supervision (equal), visualization (lead), writing – original draft (equal), writing – review and editing (lead). **Amane Noi:** data curation (equal), formal analysis (equal), investigation (lead), project administration (supporting), visualization (equal), writing – original draft (lead), writing – review and editing (equal). **Hiroto Kageyama:** visualization (equal), writing – original draft (equal), writing – review and editing (equal). **Mikiko Ueda:** conceptualization (equal), data curation (equal), investigation (equal), project administration (lead), supervision (lead), visualization (equal), writing – original draft (equal), writing – review and editing (equal). **Yoshiko Hashii:** conceptualization (equal), project administration (equal), resources (equal), visualization (equal), writing – original draft (equal), writing – review and editing (equal). **Kenji Ikeda:** data curation (lead), supervision (equal), visualization (equal), writing – original draft (equal), writing – review and editing (equal).

## Ethics Statement

This study was conducted in accordance with the ethical principles for medical research outlined in the Declaration of Helsinki, 1964, and per subsequent revisions and approved by Ethical Review Board Osaka University Hospital (IRB Approval Code no. 19410) and Clinical Research Ethics Review Committee of Osaka University Graduate School of Pharmaceutical Sciences and School of Pharmaceutical Sciences (IRB Approval Code no. 2019‐24).

## Consent

Because of the retrospective nature of the study, the need for informed consent was waived by the same ethical review boards (“Ethical Review Board Osaka University Hospital” and “Clinical Research Ethics Review Committee of Osaka University Graduate School of Pharmaceutical Sciences and School of Pharmaceutical Sciences”). A description of the study, details on the opt‐out process, and an assurance that opting out would not result in any disadvantages were publicly available on the research facility's website.

## Conflicts of Interest

The authors declare no conflicts of interest.

## Data Availability

All data generated or analyzed during this study are included in this published article.
